# Stigmatising attitudes of undergraduates towards their peers with depression: a cross-sectional study in Sri Lanka

**DOI:** 10.1186/s12888-015-0523-9

**Published:** 2015-06-19

**Authors:** Santushi D. Amarasuriya, Anthony F. Jorm, Nicola J. Reavley, Andrew J. Mackinnon

**Affiliations:** 1 Behavioural Sciences Stream, Faculty of Medicine, University of Colombo, No. 25, PO Box 271, Kynsey Road, Colombo 8, Sri Lanka; 2 Centre for Mental Health, Melbourne School of Population and Global Health, 207 Bouverie Street, University of Melbourne, Melbourne, VIC 3010 Australia; 3 Orygen, The National Centre of Excellence in Youth Mental Health, University of Melbourne, Parkville VIC 3052 Melbourne, Australia

**Keywords:** Stigma, Undergraduate, Depression, Mental illness, Exploratory structural equation modelling, Attitudes

## Abstract

**Background:**

There is limited research examining mental health-related stigma of undergraduates in non-western developing countries. This study examined stigma of undergraduates in Sri Lanka towards another depressed undergraduate.

**Methods:**

A hypothetical vignette of an undergraduate suffering from depression was presented. A total of 4650 undergraduates responded to scales assessing their personal stigma towards and desire for social distance from this individual. Exploratory structural equation modelling (ESEM) was performed to determine the dimensionality and loading pattern of the items on these two stigma scales. Multiple linear regressions were used to explore correlates of the identified dimensions of stigma.

**Results:**

Previous findings that the Social Distance Scale forms a single dimension and that the Personal Stigma Scale consists of two dimensions were supported. However, the measurement structure of the dimensions of stigma on the latter scales, labelled ‘Weak-not-Sick’ and ‘Dangerous-Undesirable’ , differed from previous work. A high level of stigma in relation to the ‘Weak-not-Sick’ Scale was observed. However, some correlates associated with lower levels of stigma on this scale, such as being in the Medical Faculty, were associated with higher levels of stigma on the ‘Dangerous-Undesirable’ and ‘Social Distance’ scales. In contrast, labelling the problem as a mental health-related problem, with absence of specific psychiatric terminology, was associated with lower levels of stigma on these latter two scales. Exposure to a mental health problem in family or friends or from personal experience was also associated with lower stigma on the Social Distance Scale. However, the effect sizes of these relationships were small.

**Conclusions:**

The findings highlight differences in the measurement structure and score distribution of the ‘Weak-not-Sick’ and ‘Dangerous-Undesirable’ scales when used in different cultural and demographic contexts. The dimensionality of stigma relevant to these scales must always be established prior to their use in different contexts. Furthermore, campaigns targeted at improving knowledge about depression as a real illness and as a psychiatric condition need to ensure that such attempts are not associated with increases in other aspects of stigma.

**Electronic supplementary material:**

The online version of this article (doi:10.1186/s12888-015-0523-9) contains supplementary material, which is available to authorized users.

## Background

The high prevalence of depression worldwide in youth and in particular, in undergraduate students, highlights the need to investigate their help-seeking behaviour and factors that may act as barriers to their help-seeking [1, 2]. One such factor is the stigma or negative attitudes that a young person or undergraduate has towards people with mental disorders. This has been demonstrated to influence perceived need for help and intentions to seek help, beliefs about the sources of help to approach and actual treatment use [3–5].

Although there is a range of factors that affect the attitudes of undergraduates towards mental illness, and more specifically, towards depression, findings regarding their effects are not unequivocal. For example, there is conflicting evidence about differences in stigmatising attitudes as a function of gender and age [3, 6–8]. Findings about how stigma is affected by exposure to mental health problems, such as through course of study (e.g., medical or health-related disciplines) [8–12], personal experience [13–15] and the experiences of family member and friends [7, 12, 13, 15, 16], are also not consistent. Studies have also examined the effects that labelling a disorder can have on related stigmatising attitudes in undergraduates. Although some show that using psychiatric terminology is associated with higher levels of stigma [17, 18], the absence of such effects has also been observed [6]. Thus, there is no clear picture of the predictors of stigmatising attitudes in undergraduates.

One reason for these differences is that stigma is a multidimensional construct [19–21], and various studies have examined different dimensions of stigma which have different correlates. Another reason for such differences could be the variations in the cultural or socio-political contexts of these studies [3, 22, 23]. Hence, further examination of depression-related undergraduate stigma in differing cultures would provide a more global understanding of this construct. Much of the research done thus far has focussed on western or developed countries, pointing to the need for more work in less well-researched, non-western developing contexts.

In a review of stigmatising attitudes towards the mentally ill in Asian developing countries such as Sri Lanka, stigmatising attitudes were highly evident, although related research in these countries was limited in scope [24]. One study found that depression-related stigma among medical students and doctors in Sri Lanka is higher than among their British counterparts [25]. In contrast, the Sri Lankan undergraduates had lower stigmatising attitudes about the unpredictability and dangerousness of those with schizophrenia. However, this finding contradicted the findings of a similar comparison between Sri Lankan and British samples [26]. Carers of patients with depression also held stigmatising attitudes towards their care-receivers, such as that the problem is a sign of personal weakness and that the person is more likely to be violent than a person in the community [27]. However, desire for social distance from the mentally ill was low in the general population when examined in the National Mental Health Survey of Sri Lanka using qualitative methodology [28]. A range of limitations in previous studies, including varying ways of assessing stigma, non-representative sampling of undergraduates and small samples, indicate the need for more work in this area. Hence, the present study aimed to examine depression-related stigmatising attitudes in undergraduates in Sri Lanka, in particular, their personal stigma as measured by the Depression Stigma Scale [29] and willingness for contact with those with depression as measured by the Social Distance Scale [30]. The factor structures of these scales have already been examined in Australia [19, 31] and Japan [32]. We therefore aimed to carry out a similar examination in Sri Lanka using the same exploratory structural equation modelling approach. This also enabled an assessment of the cross-cultural applicability of these scales. Predictors of the identified dimensions of stigma were also examined to gain an understanding of the correlates of stigma in undergraduates in Sri Lanka.

## Method

### Design, participants and setting

This was a cross-sectional study in which data were collected from June to November 2013, from undergraduates in all years of study at five of the six faculties of the University of Colombo; the Faculties of Arts, Law, Management and Finance, Medicine and Science as well as the University of Colombo School of Computing. As those in the second and third years of the Faculty of Education attend lectures at the Faculty of Arts, it was assumed that these students would also be represented during data collection.

### Questionnaire

This study was a part of a larger Depression Literacy Survey. The measure underwent several stages of adaptation, including review by mental health professionals in Sri Lanka, to be suitable for use among the undergraduate population. The questionnaire was a dual-language measure presented in two versions; one in English-Sinhala and the other in English-Tamil, where questions were presented in English in both versions and participants were able to use the version with their preferred translation (see Additional file 1 for the English-Sinhala version of the questionnaire).

Subsequent to questions regarding socio-demographic information, the questionnaire presented a vignette of an undergraduate named “Z”, with Moderate Depression as per the Diagnostic and Statistical Manual of Mental Disorders -IV. Participants were asked to imagine that this undergraduate was of their own age and gender.

The vignette was as follows:

‘Z’ has been feeling unusually sad and miserable for the last few weeks. Even though ‘Z’ feels tired all the time ‘Z’ has difficulty falling asleep almost every night. ‘Z’ doesn’t feel like eating and has lost weight. ‘Z’ finds it difficult to concentrate on studies and ‘Z’s marks have dropped. ‘Z’ complains of feeling lifeless and finds even day to day tasks too much to handle. ‘Z’ finds it difficult to make decisions even about minor matters. ‘Z’ doesn’t want to go to university and tries to stay alone all the time. ‘Z’ seems very different to what ‘Z’ was like before. ‘Z’s’ parents and friends are very worried about ‘Z’.

Subsequent to this, participants were presented with three open-ended questions assessing their ability to recognise the problem, intentions to seek help if personally affected by it and intended actions to help ‘Z’; scales assessing their beliefs about help-seeking options for the problem; questions examining their exposure to the problem through family or friends or through personal experience; the Patient Health Questionnaire-9 (PHQ-9) for depression (with Sinhala/ Tamil validated versions [28]); and a measure of exposure to stressful life events. The following is a description of the personal stigma and social distance scales that were also part of the questionnaire, the findings of which were the focus of this study.

#### Personal stigma scale

The scale was based on the Depression Stigma Scale [29] which was adapted for use with young people [19]. The phraseology of a few items was adapted to capture the intended content in light of the common parlance of the study population and translation languages used. The personal stigma items were: (1) ‘Z’ could make the problem just go away if ‘Z’ wanted to (2) ‘Z’s problem is a sign of personal weakness (3) ‘Z’s problem is not a real illness (4) ‘Z’ could be dangerous to others (5) It is best to avoid ‘Z’ so that you don’t develop this problem yourself (6) ‘Z’s problem makes ‘Z’s behaviour unpredictable (7) You would not tell anyone if you had a problem like ‘Z’s. The items were rated on a five point scale with the rating options ‘strongly agree’ , ‘agree’ , ‘neither agree nor disagree’ , ‘disagree’ and ‘strongly disagree’. Items were reverse scored so that higher scores indicated greater stigmatising attitudes.

#### Social distance scale

This scale measured willingness to have social contact with the person described. It is based on a social distance scale for youth [19] which was adapted from one for adults [30]. The phraseology of a few of the items was adapted to be suitable for use among the study population. The items were rated for willingness to (1) go out with ‘Z’ in the weekend (2) do joint study with ‘Z’ (3) invite ‘Z’ to your house (4) go to ‘Z’s house (5) develop a close friendship with ‘Z’. Items were rated on a four point scale with the rating options ‘yes, definitely’ , ‘yes, probably’ , ‘probably not’ and definitely not’ with higher scores indicating greater desire for social distance.

### Procedure

The questionnaire was administered at lectures. During distribution of the questionnaires, the potential participants were given a brief introduction to the study and informed that participation was voluntary. In most instances this was done by SDA and in her absence, this information was read out by the relevant lecturer. Students were then referred to the participant information sheet that was attached to the questionnaire, providing more details about the study. Participants took approximately 20 min to complete the questionnaire.

#### Coding responses for problem recognition

Reponses to the open-ended question relating to problem recognition were coded using the coding categories used in similar studies as a guideline [33, 34]. However, as such problem recognition work had not been done previously among this undergraduate population, the coding categories were created for all responses which varied in meaning. Responses were coded in relation to each category, with some being relevant to multiple categories. Coding was done by SDA, a clinical psychologist trained in Sri Lanka, who is fluent in English and Sinhala, the languages used by most participants. SDA coded the Tamil responses using the translations provided by a professional translator. Subsequent to this, the authors identified the common themes that emerged in the coding categories, with the final categories being those nominated by ≥ 5 % of the respondents or approximating correct recognition of the condition. The coding categories included ‘depression’ , ‘mental illness’ , ‘mental issue’ (such as *mental problem, mental unrest, mentally in a mess, mental break down*), ‘stress, pressure, mental suffering’ , ‘university/ education related problems’ , ‘romantic relationship related problems’ , with all other responses assigned to an ‘other’ category. Stigma was examined in relation to recognition of the problem as ‘depression’ , and by the use of a mental health-related label that did not contain psychiatric terminology. In light of problem recognition being considered in relation to these two mutually exclusive categories and some participants’ responses being relevant to more than one category, the responses were recoded using a hierarchy. If participants mentioned both ‘depression’ and another mental health-related label not involving psychiatric terminology (responses relevant to the categories ‘mental issue’ and ‘stress, pressure and mental suffering’), they were coded as responding with ‘depression’. Similarly, if participants responded with mental health-related labels, both with psychiatric terminology (e.g., ‘mental illness’) and without psychiatric terminology, they were coded as responding with the former. In other words, participants’ responses were coded in terms of the most specific psychiatric label they used.

### Ethics approval

Approval was obtained from the Ethics Review Committees of the Faculty of Medicine, University of Colombo and the University of Melbourne.

### Statistical analysis

Items on the stigma scales were analysed using percent frequencies and 95 % confidence intervals.

Exploratory structural equation modelling (ESEM) was performed to identify the factor structure of the scales used. ESEM was preferred over confirmatory factor analysis (CFA). CFA requires fixed binary (present/absent) patterns of loadings on the factors. This can lead to poor model-fit and inflated correlations between factors and other variables in the models [35]. ESEM allows for the items to load freely on the identified factors rather than being pre-specified or inappropriately forcing loadings to zero [36].

A model with a single factor for the personal stigma items was not an acceptable fit. Hence, in line with previous findings [19, 31, 32], ESEM specified two exploratory factors on which all items loaded freely, rotated to an oblique Geomin algorithm for these items. The model also included a single confirmatory factor for the social distance items with all items specified to load on this factor. This specification, coupled with examination of residuals, enabled the testing of whether social distance was a separate construct, albeit one related to the stigma factors. All factors were permitted to correlate with other factors identified in the model. Item responses were treated as ordinal data, with polychoric correlations estimated between items. Model parameters were estimated using a robust Weighted Least Squares Method with Diagonal Weight Matrix (WLSMV) in Mplus 7.2 [36].

As dual-language measures were utilised, with a total of three languages used in the questionnaire, ESEM was also used to establish measurement invariance for the two forms of the scales. Given the dual-language nature of the measures, it was not possible to determine which language a participant used when reading the stigma items. Hence, the language used by participants to answer the three open-ended questions in the Depression Literacy Survey measure (problem recognition, help-seeking intentions, and intended actions to help ‘Z’) was considered an indicator of their dominant or preferred language option. Participants who answered all three open-ended questions in either English or Sinhala were included in the analysis. The low number of Tamil responses did not permit inclusion of these in this analysis.

Separate multivariable linear regressions were used in relation to each of the stigma scale scores (dependent variables) to examine which of the following dummy coded variables (independent variables) predicted the stigma scores: gender, faculty, year of study, age category, residence, religion, if problem in vignette was experienced by family or friends, if problem was experienced personally, having Major Depression as per the PHQ-9 [37], recognising the problem in the vignette as depression and recognising it as a mental health-related problem in the absence of psychiatric terminology.

## Results

Almost all of those who were approached for the survey participated, with a total of 4671 survey responses. Mplus can accommodate missing responses, so questionnaires were considered as valid for analysis if there were responses for any of the items on either of the stigma scales, leading to a total of 4650 responses. From these, 96.4 % participants had responded to all items on the two stigma scales. 96.0 % of responses were in the English-Sinhala version and the rest in the English-Tamil version. This sample was approximately 52 % of the total undergraduate population of the University of Colombo. Table 1 provides the demographic and other individual characteristics of the participants.

**Table 1:** Socio-demographic and other individual characteristics of undergraduate sample

Variables	n	Percentage
Socio-demographic variables		
Gender		
Male	1439	30.9
Female	3207	69.0
Faculty of Study		
Arts and Education^a^	1189	25.6
Law	613	13.2
Management and Finance	1025	22.0
Medicine	615	13.2
Science	683	14.7
School of Computing	524	11.3
Year of study		
1^st^ year	1936	41.6
2^nd^ year	1236	26.6
3^rd^ year	834	17.9
4^th^ year	530	11.4
5^th^ year^b^	114	2.5
Age		
Mean = 22.18 years (SD = 1.46)		
18- 20 years	515	11.1
21- 23 years	3336	71.7
24 and above	791	17.0
Place of residence while going to university		
Home	1743	37.5
Hostel	1396	30.0
Rented place	1184	25.5
Home of friend/ relative	271	5.8
Other (or more than two of the above)	51	1.1
Province of residence(longest duration)		
Western province (province to which the University belongs)	2159	46.4
Other provinces	2462	52.9
Ethnicity		
Sinhala	4265	91.7
Tamil	189	4.1
Sri Lankan Moor	146	3.1
Burgher, Malay, Other	46	1.0
Religion		
Buddhist	4048	87.1
Hindu	157	3.4
Islam	151	3.2
Roman Catholic	215	4.6
Other	73	1.6
Other variables		
Problem in vignette experienced by family/ friends		
Yes	1695	36.5
No	1773	38.1
Don’t know	1053	22.6
Problem in vignette experienced personally		
Yes	1510	32.5
No	2525	54.3
Don’t know	325	7.0
Screening positive for Major Depression as per the PHQ-9		
Yes	401	8.6
No	4249	91.4
Recognition of problem in vignette as ‘depression’		
Recognised	790	17.0
Not recognised	3728	80.2
Recognition of problem in vignette as a mental health-related problem; but psychiatric terminology not used		
Recognised	2341	50.3
Not recognised	2177	46.8

^a^Those in the Faculty of Education only comprised 5.6 % of this group

^b^The 5^th^ year only included students in the Faculty of Medicine

### Personal stigma and desire for social distance

Items on the Personal Stigma Scale were recoded as binary responses to indicate a participant’s agreement/disagreement with these items and are presented in Table 2. Similarly, items on the Social Distance Scale were recoded as binary responses to indicate the percentage of participants willing/ unwilling to interact with ‘Z’ and are presented in Table 3.

**Table 2:** Percentage of undergraduates (with 95 % CIs) showing agreement/disagreement with statements about depression-related personal attitudes (n = 4588-4628)

Statement	Agreement	Disagreement
1. could make problem go away	69.4 (68.1-70.7)	16.1 (15.1-17.2)
2. problem is a sign of personal weakness	50.8 (49.3-52.2)	24.3 (23.0- 25.5)
3. problem is not a real illness	54.4 (53.0-55.8)	22.4 (21.2-23.6)
4. could be dangerous to others	17.6 (16.5-18.7)	53.3 (51.9-54.7)
5. avoid ‘Z’ to prevent developing problem in oneself	4.9 (4.3- 5.5)	90.1 (89.2-91.0)
6. problem makes behaviour unpredictable	66.8 (65.5-68.2)	12.9 (11.9-13.9)
7. would not tell anyone if you had problem	7.9 (7.2-8.7)	79.7 (78.5-80.8)

**Table 3:** Percentage of undergraduates (with 95 % CIs) willing/unwilling to socially interact with ‘Z’ (n = 4588-4609)

Statement	Unwilling	Willing
1. go out with ‘Z’ in the weekend	8.8 (8.0- 9.7)	91.2 (90.3- 92.0)
2. do joint study with ‘Z’	12.2 (11.3-13.2)	87.8 (86.8- 88.7)
3. invite ‘Z’ to your house	10.7 (9.8- 11.6)	89.3 (88.4- 90.2)
4. go to ‘Z’s house	14.5 (13.5- 15.5)	85.5 (84.5- 86.5)
5. develop a close friendship with ‘Z’	6.8 (6.0- 7.5)	93.2 (92.5- 94.0)

### Exploratory structural equation models

Additional file 2 shows the effect on model fit of introducing additional constraints on between-group model parameters in order to achieve measurement invariance for the two language groups, English and Sinhala. The basic ‘Configurational’ form fits the same structure to each group but allows all model parameters to differ between them. Essentially, this amounts to fitting the models to each group separately, but deriving combined goodness of fit statistics. This model had a significant chi-square value, but all other indices of goodness of fit implied that the model was an excellent fit to the data. The size of the chi-square can be seen as reflecting the large sample size rather than a substantial lack of fit. Metric and Scalar models incrementally constrain item factor loadings and response category thresholds between groups. The effect of constraining factor loadings was negligible. While the impact of equating the item thresholds was larger, overall model fit remained excellent, implying that the factor structure is invariant across the two groups. The final ‘Structural’ model constrained residual variance to equality. This is not necessary for invariance, but demonstrates that this model fits well (as seen in Additional file 2) reinforcing the notion that the items function in an equivalent manner in each group.

As strict measurement invariance could be demonstrated, the factor loadings shown in Table 4 are those estimated by combining the two groups. The clearest results were given by the social distance items which all show strong, uniform loadings on a single factor. With regard to the two exploratory factors, the factor labelled as ‘Weak-not-Sick’ was very clearly defined by three relevant items, with the other items loading on this only negligibly. The second factor had high loadings for items endorsing the individual as being dangerous and best avoided, as well as for the item regarding self-disclosure. Critically, the item describing the individual as unpredictable had only a very low loading on this factor (as well as on the other factor). Hence the factor was labelled as ‘Dangerous-Undesirable’ to reflect the aspects of stigma measured by the items. Table 5 shows that these two dimensions of stigma are almost uncorrelated as are the Social Distance and the Weak-not-Sick factors. There was a moderate correlation between the Social Distance and Dangerous-Undesirable factor.

**Table 4:** Factor loadings

Item	Loadings		
	Weak-not-Sick	Dangerous-Undesirable	Social Distance
Personal Stigma			
1. could make the problem just go away	0.59	0.00	—
2. problem is a sign of personal weakness	0.63	0.12	—
3. problem is not a real medical illness	0.55	-0.10	—
4. could be dangerous to others	-0.01	0.41	—
5. avoid ‘Z’ to prevent developing problem in oneself	0.02	0.64	—
6. problem makes behaviour unpredictable	0.07	0.12	—
7. would not tell anyone if you had problem	-0.04	0.47	—
Social Distance			
1. go out with ‘Z’ in the weekend	—	—	0.74
2. do joint study with ‘Z’	—	—	0.72
3. invite ‘Z’ to your house	—	—	0.89
4. go to ‘Z’s house	—	—	0.86
5. develop a close friendship with ‘Z’	—	—	0.80

**Table 5:** Factor correlations, correlations of scale scores with factor scores and scale reliabilities

		Personal stigma		Social Distance
		Weak-not-Sick	Dangerous-Undesirable	
Personal – Weak-not-Sick		—	0.09^**^	-0.08^**^
Personal – Dangerous-Undesirable		0.18^***^	—	0.23^**^
Social Distance		-0.14^***^	0.38^***^	—
Reliability	r_scale~factor_	0.98^**^	0.90^**^	0.97^**^
	Cronbach’s α	0.56	0.38	0.84

^**^p < .001; ^***^ *p* < .0001

Notes Factor correlations below diagonal; scale correlations above

r_scale~factor_ = correlations of scale scores with factor scores

### Scale scores

As the factor structures of the Personal Stigma and Social Distance scales were similar in the Sinhala and English language groups and comparable to previous findings, it was considered acceptable to construct scales derived from these factors for the full sample, including those responding in Tamil. As with previous research [31, 32], factor scores estimated by Mplus were compared to corresponding scale scores calculated by summing items with substantial loadings (> .30) on each factor. Table 5 shows that the scale scores were very highly correlated with factor scores and had a similar pattern of interrelations as their underlying, latent variables. However, conventional reliability analyses showed that while the internal consistency of the Social Distance Scale was high, that of the two stigma scales was low and unacceptable by conventional standards. Nevertheless, the high correlation between the scale scores and factor scores provided support that they were tapping distinct dimensions of stigma and that further analyses pertaining to these scales were defensible.

Scale scores were preferred over factor scores as the latter limits application of the current work by other researchers. Each participant was permitted to have a single response missing for the Social Distance Scale, with the missing score prorated using the mean of existing item scores (36 occurrences). Missing responses were not permitted for the other scales due to the low number of scale items.

**Fig. 1:**
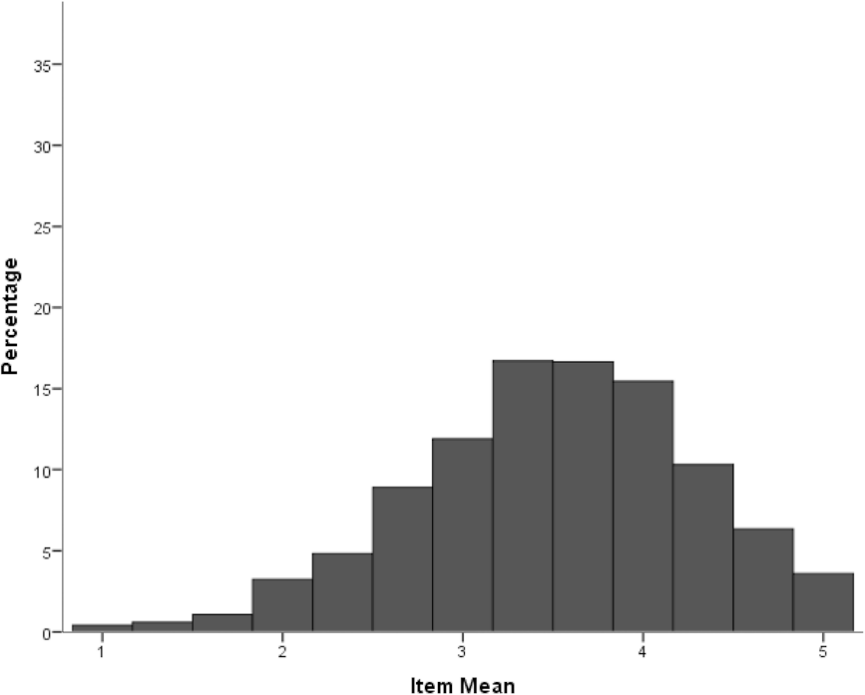
Distribution of Weak-not-Sick Scale scores (item means)

**Fig. 2:**
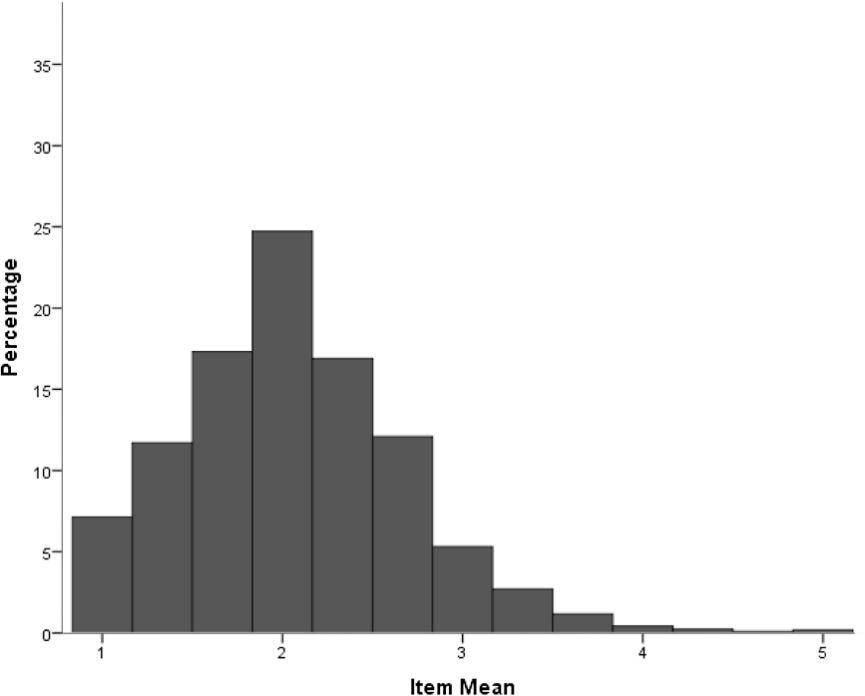
Distribution of Dangerous-Undesirable Scale scores (item means)

**Fig. 3:**
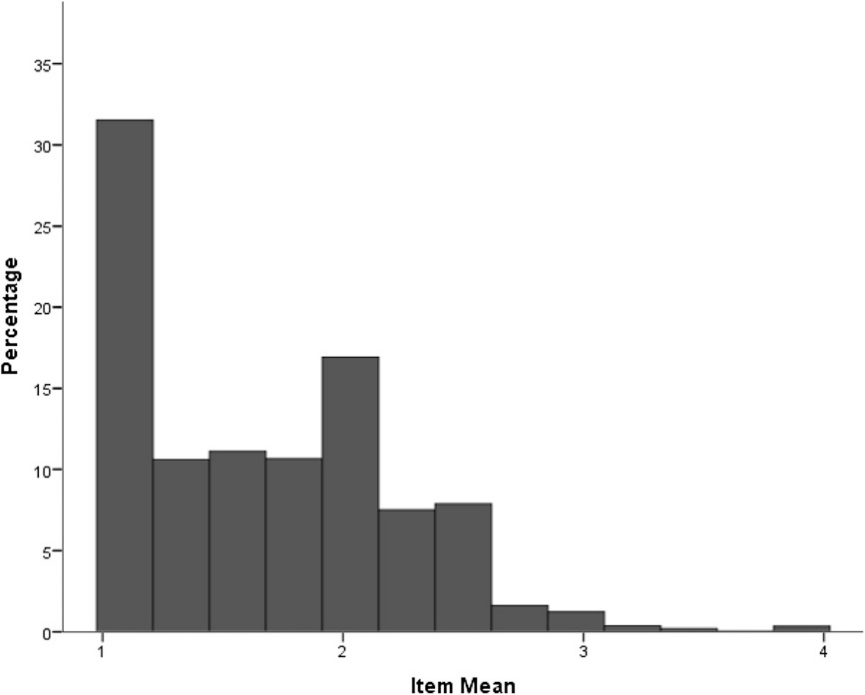
Distribution of Social Distance Scale scores (item means)

As seen in Fig. 1, the distribution of item-ratings of the Weak-not-Sick Scale was negatively skewed with a more stigmatised modal rating. In contrast, as seen in Figs. 2 and 3 respectively, the item- ratings for both the Dangerous-Undesirable and Social Distance scales were positively skewed with more participants reporting non-stigmatising attitudes.

### Predictors of scale scores

Table 6 presents the means of the scale scores and the relationships between these scores and the examined variables, in relation to the dummy coded reference groups. Higher scores on the Weak-not-Sick Scale were associated with being female or a Hindu (compared to Buddhist), while lower scores were associated with being in the Medical Faculty compared to all other faculties (except Law), a 2^nd^ year student or a 5^th^ year Medical student (compared to 1^st^ years), in the 21–23 age category (compared to being 18–20 years), in the ‘other’ religion category (compared to Buddhists), uncertain if the problem in the vignette was personally experienced (compared to not having such an experience) and recognising this problem as depression or a mental health-related problem, where psychiatric terminology was not used.

**Table 6:** Examination of predictors of scale scores using multivariable linear regression

	Weak-not-Sick Scale		Dangerous-Undesirable Scale		Social Distance Scale	
	(*N* = 3858) (scale mean = 3.51; SD = 0.78)		(*N* = 3864) (scale mean = 2.06; SD = 0.63)		(*N* = 3872) (scale mean = 1.67; SD = 0.54)	
	Standardised regression coefficient (95 % CI)		Standardised regression coefficient (95 % CI)		Standardised regression coefficient (95 % CI)	
Gender (reference group: Male)						
Female	0.05^**^	(0.02, 0.08)	-0.12^***^	(-0.15, -0.09)	-0.001	(-0.03, 0.03)
Faculty (reference group: Medicine)						
Arts and Education	0.12^***^	(0.07, 0.18)	-0.18^***^	(-0.23, -0.12)	-0.28^***^	(-0.33, -0.22)
Law	0.02	(-0.03, 0.06)	-0.19^***^	(-0.24, -0.14)	-0.20^***^	(-0.25, -0.16)
Management	0.12^***^	(0.07, 0.18)	-0.17^***^	(-0.23, -0.12)	-0.21^***^	(-0.26, -0.16)
Science	0.08^**^	(0.04, 0.13)	-0.12^***^	(-0.16, -0.07)	-0.08^**^	(-0.12, -0.03)
Computer	0.07^**^	(0.03, 0.12)	-0.12^***^	(-0.16, -0.07)	-0.15^***^	(-0.19, -0.10)
Year (reference group: 1^st^ year)						
2^nd^ year	-0.05^*^	(-0.08, -0.01)	0.06^**^	(0.03, 0.10)	0.08^***^	(0.04, 0.12)
3^rd^ year	0.01	(-0.02, 0.05)	0.05^**^	(0.01, 0.09)	0.08^***^	(0.04, 0.11)
4^th^ year	-0.03	(-0.07, 0.02)	0.04	(-0.002, 0.08)	0.08^***^	(0.04, 0.12)
5^th^ year (Medicine)	-0.08^***^	(-0.12, -0.04)	0.01	(-0.03, 0.04)	-0.05^*^	(-0.09, -0.01)
Age category (reference group: 18-20 years)						
21-23 years	-0.05^*^	(-0.10, -0.001)	-0.002	(-0.05, 0.05)	0.002	(-0.05, 0.05)
24 and above	-0.03	(-0.09, 0.03)	0.004	(-0.06, 0.06)	-0.01	(-0.07, 0.05)
Residence (reference group: Home)						
Hostel	-0.02	(-0.06, 0.02)	-0.01	(-0.05, 0.02)	-0.05^*^	(-0.08, -0.01)
Rented place	0.00	(-0.04, 0.04)	-0.02	(-0.05, 0.02)	-0.01	(-0.04, 0.03)
Home of friend/ relative	-0.01	(-0.04, 0.02)	0.01	(-0.02, 0.04)	0.03^*^	(0.00, 0.06)
Other	0.01	(-0.02, 0.04)	-0.01	(-0.04, 0.02)	0.01	(-0.02, 0.04)
Religion (reference group: Buddhist)						
Hindu	0.04^**^	(0.01, 0.07)	0.03	(-0.001, 0.06)	-0.01	(-0.04, 0.02)
Islam	-0.005	(-0.04, 0.03)	0.01	(-0.02, 0.04)	-0.04^**^	(-0.07, -0.01)
Roman Catholic	-0.02	(-0.05, 0.01)	0.02	(-0.01, 0.06)	0.02	(-0.01, 0.05)
Other	-0.04^**^	(-0.07, -0.01)	0.03	(-0.005, 0.06)	-0.03^*^	(-0.06, -0.001)
Problem in vignette experienced by family or friends (reference group: response: No)						
Response: Yes	-0.01	(-0.04, 0.03)	0.02	(-0.02, 0.06)	-0.04^*^	(-0.08, -0.005)
Response: Don’t know	-0.003	(-0.04, 0.03)	0.02	(-0.01, 0.06)	0.07^***^	(0.04, 0.10)
Personal experience of problem in vignette (reference group: response: No)						
Response: Yes	-0.01	(-0.05, 0.02)	-0.01	(-0.04, 0.03)	-0.05^**^	(-0.09, -0.02)
Response: Don’t know	-0.05^**^	(-0.08, -0.02)	0.004	(-0.03, 0.04)	0.02	(-0.02, 0.05)
Screening positive for Major Depression as per the PHQ- 9 (reference group: No)						
Yes	-0.02	(-0.05, 0.01)	0.06^***^	(0.03, 0.09)	0.01	(-0.02, 0.04)
Problem in vignette recognised as depression (reference group: Not recognised)						
Recognised	-0.13^***^	(-0.17, -0.09)	0.01	(-0.02, 0.05)	-0.02	(-0.06, 0.02)
Problem in vignette recognised as a mental health-related problem; but psychiatric terminology not used (reference group: Not recognised)						
Recognised	-0.06^**^	(-0.10, -0.03)	-0.04^*^	(-0.08, -0.01)	-0.06^**^	(-0.10, -0.02)
R square	.065		.067		.081	
Adjusted R square	.058		.061		.074	

^*^ *p* > .05; ^**^ *p* > .01; ^***^ *p* > .001

Higher scores on the Dangerous-Undesirable Scale were associated with being in the Medical Faculty, being a 2^nd^ or 3^rd^ year student (compared to a 1^st^ year) and screening positive for Major Depression on the PHQ-9. Lower scores were associated with being female and recognising the problem in the vignette as a mental health-related problem (psychiatric terminology not used).

For the Social Distance Scale, higher scores were associated with being in the Medical Faculty, being a 2^nd^, 3^rd^ or 4^th^ year student (compared to a 1^st^ year), residing in the home of a friend/ relative (compared to home), and being uncertain whether the problem had been experienced by one’s family or a close friend (compared to the problem not being experienced by them). Lower scores were associated with being a 5^th^ year Medical student (compared to being a 1^st^ year student), living in a hostel (compared to home), the Islamic faith and the ‘other’ religion category (compared to being Buddhist), personally experiencing the problem or one’s family or close friend experiencing it (compared to the absence of such experiences), and recognising the problem as a mental health-related problem (psychiatric terminology not used).

## Discussion

This study examined depression-related stigma of undergraduates in Sri Lanka using the personal stigma component of the Depression Stigma Scale [19, 29] and the Social Distance Scale [19, 30]. While the latter scale consisted of a unitary dimension, the Personal Stigma Scale consisted of two dimensions. Analyses of the predictors of the scale scores found different combinations of predictors for the different scales, with faculty of study being one of the strongest predictors across the different scales. Following is a more in-depth discussion of these findings.

### Structure of stigma scales

ESEM replicated previous findings that the Social Distance Scale measures a distinct dimension of stigma [19, 31, 32], supporting its usability in different cultural contexts. Findings also support previous work that the Personal Stigma Scale does not consist of a unitary dimension but two distinct dimensions. However, the two exploratory factors differed somewhat from findings in previous studies. Although the structure of the Weak-not-Sick dimension was similar to what had been observed, our study differed in relation to the measurement structure of the other dimension which had been previously labelled as ‘Dangerous-Unpredictable’. We found that the item regarding unpredictability did not fit within the measurement structure that was identified for this dimension. This led to the re-evaluation of this factor in this sample, suggesting that it involves perceptions of dangerousness and undesirability. Furthermore, the item about avoidance loaded on this factor, differing from findings from Australia but being similar to the findings of the Japanese study. It is postulated that the perception of contagion of depression measured by this item aligns more with this ‘Dangerous-Undesirable’ dimension than the ‘Weak-not-Sick’ dimension.

It is of concern that the internal consistencies of both the Weak-not-Sick and Dangerous-Undesirable scales were low, being more so for the latter. While this may reflect the small number of items comprising each scale, it also reflects the slightly lower factor loadings observed in this sample compared to previous studies. However, the use of Cronbach alpha to assess a measure’s reliability is being questioned [38, 39] with structural equation modelling considered to provide a more accurate estimate [40, 41].

### Stigma in undergraduates in relation to the stigma scales

The mean of the Weak-not-Sick Scale (Table 6) and the percentage of those endorsing stigmatising attitudes on this scale (Table 2) were higher than seen in Australia and Japan [31, 32]. This indicates that this population had a greater tendency to perceive the onus of the illness and its trajectory to be on the individual. This aligns with previous findings of Sri Lankans possessing higher blaming attitudes than their British counterparts towards the mentally ill [25]. It is also consistent with an emerging pattern of blaming attitudes towards the mentally ill in Asian populations [25], with undergraduates perceiving depression to be a personal weakness or failure and a problem the person can get rid of or be blamed for [22, 42–44]. While reluctance to seek professional treatment is seen among undergraduates across many contexts [45, 46], such stigmatising attitudes about depression in these Asian contexts could further impede the treatment of those who are depressed.

It is encouraging that stigma as per the Social Distance Scale (Tables 3 and 6) was generally low among the undergraduates in comparison to the previous Japanese and Australian studies. The existence of lower levels of stigma in Sri Lanka for this dimension is supported by previous research which found that Sri Lankans’ desire for social distance from those with schizophrenia was less than the British, despite their overall stigma towards these individuals being higher [26]. These findings can be interpreted in relation to Waxler’s [47] account of the Sri Lankan (Sinhalese) society in which it is the family and not the individual that is the basic unit of society, where the family or larger social network does not alienate the mentally ill but considers it their responsibility to act collectively to assist these persons to re-integrate into society. This ideology might be an advantage for the recovery of the mentally ill in Sri Lanka, given the short supply of mental health services [48] and the demands placed on the family or close social network as care providers. Stigma on the Dangerous-Undesirable Scale was also low, further affirming this population’s tolerance of those with depression. In contrast, ratings of unpredictability of the person were high, being the most endorsed stigmatising attitude. A similar pattern of attitudes was seen among medical students and doctors [25] indicating the need for more research on this aspect of stigma.

### Predictors of depression-related stigma

Findings indicate that male undergraduates have more negative attitudes about their depressed peers relating to the Dangerous-Undesirable dimension. Such perceptions might stem from mental illness being still perceived as more taboo for males [49]. However, when considering the Weak-not-Sick dimension, females had a greater tendency to perceive the problem as a “weakness” and not a “sickness”, which might be associated with the perception that depressive symptomatology align with female expressions of negative emotionality. It would be interesting to explore further, if such differences in the findings are in fact influenced by gender-specific perceptions and expectations of the disorder and its behavioural manifestation. Furthermore, an ethnographic exploration of the effects of other socio-demographic characteristics on stigma would enable a better understanding of differences in stigma within the population. For example, although the lower scores for the Social Distance Scale for those of the Islamic faith might be explained by descriptions about the lower prevalence of social stigma in Islamic societies, which believe that the mentally ill are a communal responsibility [50], more evidence for the relevance of such explanations in the target population is needed.

There was some evidence that being in a higher year of study (compared to the first-year), considered akin to a greater level of maturity and exposure to depression-related knowledge, was associated with a higher likelihood of perceiving the problem as a “real illness”. However, this was sometimes associated with stronger attitudes about the dangerousness and undesirability of the person and unwillingness to interact with this individual. A similar trend was seen when considering faculty of study as a predictor. Being in the Medical Faculty was associated with a higher likelihood of perceiving the problem as a “real illness” and not a “weakness” and could be explained by the exposure that these students have to information about depression and medicalisation of the problem in their field of study. However, they had higher stigmatising attitudes on the Dangerous-Undesirable and Social Distance scales, indicating that medicalisation of the problem might be associated with other stigmatising attitudes towards those with depression.

It was also seen how the participants’ ability to label the condition as either depression or as a mental health-related problem, were both associated with lower scores on the Weak-not-Sick Scale. However, it was only in instances in which this label did not involve medicalisation of the problem or the use of specific psychiatric terminology that lower scores on the Dangerous-Undesirable and Social Distance scales were observed.

The findings suggest caution in the design of depression literacy initiatives for this population. While providing greater knowledge about the condition as an illness is expected to trigger appropriate help-seeking, such initiatives need to counter any negative effects this might have on other dimensions of stigma. In such approaches, mere provision of knowledge about depression in an academic sense, unaccompanied by personal understanding of affected persons, might be inadequate to dispel the misconceptions of undergraduates about the condition [51]. For example, while those in the Medical Faculty might be well versed with the symptomatology of depression, it is only later in their training that they receive more intensive clinical experience relating to depression. Hence, until such exposure, they might not have adequate experiences confronting their misconceptions about depression. Supporting this argument is evidence of a reduction of depression-related stigma among fifth year medical students in Sri Lanka subsequent to a clinical attachment in psychiatry [52]. However, contrary to this, increases in contact with such patients was not associated with a decline in stigma in other undergraduate studies, with differences in findings explained by a range of factors affecting the student’s interactions including, severity of cases, variations in the type of contact (formal vs. informal and voluntary vs. involuntary) and the attitudes held within the hospital environment [8, 11, 12]. This highlights the need for more controlled and graded exposure to such clinical situations to facilitate a decline in stigma among this population.

While incorporation of experiential-based curriculum elements supplemented by appropriate supervision might be possible in the case of the Medical Faculty, opportunities for other undergraduates to obtain such experiential learning might be limited. Furthermore, caution is needed in such attempts. For example, although a cooperative short-term activity with a former mentally ill undergraduate reduced stigma in undergraduates [53], when undergraduates with mental health treatment histories were assigned as room-mates with those with no mental health history, the latter group’s stigma towards mental health treatment users increased, highlighting that naturalistic contact alone, if not structured appropriately, might be more harmful than helpful [16]. While providing undergraduates with social contact or video-based social contact with the mentally ill as brief educational interventions to reduce their stigma has been effective, it is important that these interventions expose them to the normal lives and successes of the affected individuals [54]. Such a naturally occurring encounter would be when the problem is experienced by the undergraduate’s family or friends or by the undergraduate. Our findings support previous work [7, 15] that such instances are associated with undergraduates showing greater willingness to interact with affected persons. While this provides evidence for the effectiveness of such social contact in reducing social stigma, more work is needed to examine if these effects exist when characteristics of the aforementioned contact, such as its nature and quality of relationship between the affected person and undergraduate, vary.

Although the present study provides useful insight about stigma in undergraduates in Sri Lanka, the limitations of the findings must be considered. The cross-sectional nature of the data limits interpretations regarding the causality of stigma. Furthermore, the regression models only accounted for a small percentage of variance in stigma. The standardised regression coefficients which indicate small effect sizes for some of the examined relationships warn of the caution needed when interpreting the findings and that the clinical significance of these might be small. However, as the results indicate that there might be certain trends in stigma in relation to some of the examined variables, further examination of such relationships is recommended. The findings for the Weak-not-Sick and Dangerous-Undesirable scales must also be considered with caution given the low reliability estimates found for these scales. As this study relied on explicit measures which could have led to socially desirable responding, the use of more implicit and behavioural measures is recommended [55]. Furthermore, utilisation of qualitative methodology would enable a more in-depth understanding of stigma and the ethnographic influences on this construct. As stigma might vary in relation to the disorder examined [8, 6, 56], more work is needed to examine if such differences exist in this population.

### Conclusions

The study shows that in Sri Lanka, as in other countries, stigma is a multidimensional construct. However, there were differences in the factor structure of stigma in Sri Lanka compared to other countries, indicating the need for psychometric analysis of stigma scales before use in a given culture. The findings also have implications for efforts to reduce stigma in undergraduates in Sri Lanka. While promoting a medicalised understanding of depression may have a role in improving help-seeking, caution is required that it does not increase some aspects of stigma. It is recommended that in attempts to educate undergraduates about depression, they are also provided experiential contact with depressed persons that will help combat some of their stigmatising attitudes.

## Additional files

Additional file 1:
**English-Sinhala version of questionnaire.**

Additional file 2:
**Model fit statistics for multiple group (English/Sinhala) models.**

## Abbreviations

CFA: Confirmatory factor analysis
ESEM: Exploratory structural equation modelling
PHQ-9: Patient Health Questionnaire-9
