# Supplementary material for: Stigmatising attitudes of undergraduates towards their peers with depression: a cross-sectional study in Sri Lanka
Source: BMC Psychiatry. 2015 Jun 19;15:129. doi: 10.1186/s12888-015-0523-9 (PMC4472246; doi:10.1186/s12888-015-0523-9)
Supplement: Additional file 1: — English-Sinhala version of questionnaire. [file 12888_2015_523_MOESM1_ESM.pdf]

Problems faced by Undergraduates

උපාධි අපේක්ෂකයින් මුහුණපාන ප්‍රශ්න

This survey examines some of the problems you and those you know may be facing in life and your opinions about dealing with such problems.

Instructions

- Fill this survey on your own, without discussing it with others. Our aim is to find out about your personal opinions.
- When you are required to write your answer you could respond in either English or Sinhala or both.

(මෙම සමීක්ෂණය ඔබ හා ඔබ දන්නා අය ජීවිතයේ මුහුණපාන ප්‍රශ්න හා එම ප්‍රශ්න මුහුණපෑ යුතු ආකාරය ගැන ඔබේ අදහස් විමසයි.

<u>උපදෙස්</u>

- මෙම සමීක්ෂණය අන් අය සමඟ සාකච්ඡා නොකර ඔබම පුරවන්න. අපේ අරමුණ වන්නේ ඔබේ පෞද්ගලික මතයන් විමසා බැලීමයි.
- පිළිතුර ලිවීමට අවශ්‍ය විට ඉංග්‍රීසි හෝ සිංහල හෝ එම භාෂා දෙකෙන්ම හෝ ඔබට ප්‍රතිචාර සැපයිය හැකිය.)

Please provide the following details about yourself. (කරුණාකර ඔබ පිළිබඳ පහත තොරතුරු ලබාදෙන්න.)

1. Age (වයස) ………………

2. Gender (ස්ත්‍රී/පුරුෂ භාවය) Male (පුරුෂ) ☐ Female (ස්ත්‍රී) ☐

3. Nationality (ජාතිය) Sri Lankan (ශ්‍රී ලාංකීය) ☐ Other (වෙනත්) ☐

4. Ethnicity (ජනවර්ගය)

   Sinhala (සිංහල) ☐ Tamil (ද්‍රවිඩ) ☐ Sri Lankan Moor (ශ්‍රී ලංකා යෝනක) ☐
   Burgher (බර්ගර්) ☐ Malay (මැලේ) ☐ Other (වෙනත්) ☐ → specify (දක්වන්න)………………

5. Religion (ආගම)

   Buddhist (බෞද්ධ) ☐ Hindu (හින්දු) ☐ Islamic (ඉස්ලාම්) ☐ Christian (ක්‍රිස්තියානි) ☐
   Roman Catholic (රෝමානු කතෝලික) ☐ Atheist (ආගමක් නොඅදහන) ☐
   Other (වෙනත්) ☐ → specify (දක්වන්න)………………..

6. District you have resided in for the longest time (ඔබ වැඩිම කාලයක් පදිංචිව සිටි දිස්ත්‍රික්කය) …………………..
   duration (දළ කාලය)…………

7. Place you reside when going to university (විශ්ව විද්‍යාලයට යාමේදී ඔබ වාසය කරන ස්ථානය)

   At home (ගෙදර) ☐ Hostel (නේවාසිකාගාරය) ☐ Rented place (කුළී ස්ථානයක) ☐
   Home of friend/ relative (මිතුරු/ඥාති නිවසක) ☐ Other (වෙනත්) ☐ → specify (දක්වන්න)………………

8. Your Faculty of study (ඔබේ අධ්‍යයන පීඨය) ……………………………………………………………….......................

9. Your year of study in your course (ඔබේ පාඨමාලාවේ දැනට හදාරන වර්ෂය)

1st (1 වැනි) ☐ 2nd (2 වැනි) ☐ 3rd (3 වැනි) ☐ 4th (4 වැනි) ☐ 5th (5 වැනි) ☐

Read the case below about an undergraduate identified as 'Z'. Consider 'Z' and you to be of the same age and gender.

('Z' ලෙස හඳුන්වා ඇති උපාධි අපේක්ෂකයෙකුගේ පහත විස්තරය කියවන්න. 'Z' හා ඔබ සමාන වයසකින් හා ස්ත්‍රී-පුරුෂ භාවයෙන් යුතු යැයි සලකන්න.)

'Z' has been feeling unusually sad and miserable for the last few weeks. Even though 'Z' feels tired all the time 'Z' has difficulty falling asleep almost every night. 'Z' doesn't feel like eating and has lost weight. 'Z' finds it difficult to concentrate on studies and 'Z's marks have dropped. 'Z' complains of feeling lifeless and finds even day to day tasks too much to handle. 'Z' finds it difficult to make decisions even about minor matters. 'Z' doesn't want to go to university and tries to stay alone all the time. 'Z' seems very different to what 'Z' was like before. 'Z's' parents and friends are very worried about 'Z'.

……………………………………………………………………………..

පසුගිය සති කීපය තුළ සිට 'Z' අසාමාන්‍ය ලෙස දුකින් හා කලකිරීමෙන් පසුවෙයි. සෑම විටම 'Z'ට වෙහෙසකාරී බවක් දැනෙන නමුත් හැමදාම වාගේ 'Z'ට රාත්‍රියට නිදා ගැනීමේ අපහසුතා තිබේ. 'Z'ට ආහාර ගැනීමට රුචියක් නැති අතර බර අඩුවී ඇත. 'Z'ට අධ්‍යයන කටයුතුවලට අවධානය යොමු කිරීම අපහසු අතර 'Z'ගේ ලකුණු අඩු වී ඇත. 'Z' තමාට පණ නැති ගතියක් දැනෙන බවට පැමිණිලි කරන අතර එදිනෙදා කටයුතු පවා කරගත නොහැකි තරම් ඒවා බරක් යැයි 'Z' ට දැනේ. 'Z'ට සුළු දෙයක් ගැන වුවත් තීරණ ගැනීමට අපහසු වේ. 'Z'ට විශ්ව විද්‍යාලයට යෑමට උවමනා නැති අතර සැමවිටම හුදෙකලාව සිටීමට තැත් කරයි. 'Z' ගේ ස්වභාවය පෙරට වඩා බෙහෙවින් වෙනස් වී ඇත. 'Z' ගේ දෙමවුපියන් සහ මිතුරන් 'Z' ගැන බෙහෙවින්ම කණස්සල්ලට පත් වී ඇත.

Answer the questions based on your opinions. **(ඔබේ අදහස් පදනම් කර ප්‍රශ්නවලට පිළිතුරු දෙන්න.)**

10. What do you think is wrong with 'Z'? ('Z' ට ඇති වී තිබෙන ප්‍රශ්නය ලෙස ඔබ සිතන්නේ කුමක්ද?)

………………………………………………………………………………………………………

11. If you have this problem, what would you do? (ඔබට මෙම ප්‍රශ්නය ඇතිනම් ඔබ කරන්නේ කුමක්ද?)

………………………………………………………………………………………………………

………………………………………………………………………………………………………

………………………………………………………………………………………………………

12. Imagine that 'Z' is someone you have known for a long time and know well. You want to help 'Z'. What do you think is appropriate to do?

('Z' ඔබ දීර්ඝ කාලයක් දන්නා හා හොඳින් හඳුනන පුද්ගලයකු යැයි සිතන්න. ඔබට 'Z' ට උදව් කිරීමට අවශ්‍යයි. ඒ සඳහා ඔබ කිරීමට සුදුසු යැයි සිතන දේ කුමක්ද?)

………………………………………………………………………………………………………

………………………………………………………………………………………………………

………………………………………………………………………………………………………

13. Given below are persons and services that ‘Z’ might approach for help when dealing with this problem. Indicate how helpful/ unhelpful you think they would be.

(මෙම ප්‍රශ්නයට මුහුණදීමේදී උදව් ගැනීමට ‘Z’ ට යොමු විය හැකි පුද්ගලයින් හා සේවාවන් පහත දක්වා ඇත. ඔබේ මතය අනුව ඒවා කොපමණ උපකාරී වේද/නොවේද යන්න දක්වන්න.)

	**Person/ Service** (පුද්ගලයා / සේවාව)	**Very helpful** ඉතාමත්ම උපකාරී වේ	**Fairly helpful** තරමක් උපකාරී වේ	**Neither helpful nor unhelpful** උපකාරී හෝ අනුපකාරී නොවේ	**Fairly unhelpful** තරමක් අනුපකාරී වේ	**Very unhelpful** ඉතාමත්ම අනුපකාරී වේ	**Don’t know** දන්නේ නැත
1)	Parents (දෙමාපියන්)						
2)	Other relative (වෙනත් ඥාතියෙක්)						
3)	Boyfriend/girlfriend/ spouse (පෙම්වතා/පෙම්වතිය/සැමියා/බිරිඳ)						
4)	Friend from university (විශ්ව විද්‍යාල මිතුරෙක්)						
5)	Friend outside university (විශ්ව විද්‍යාලයෙන් පිට මිතුරෙක්)						
6)	A General Practitioner (GP) (සාමාන්‍ය වෛද්‍යවරයෙක්)						
7)	Psychiatrist (මනෝ වෛද්‍යවරයෙක්)						
8)	Psychologist (මනෝ විද්‍යාඥයෙක්)						
9)	Counsellor (උපදේශකවරයෙක්)						
10)	Organization helping people to deal with their problems (තම ප්‍රශ්න වලට මුහුණදීමට උපකාර කරන සංවිධානයක්)						
11)	Telephone counselling service (දුරකථන උපදේශන සේවයක්)						
12)	Clergy/ religious priest (ස්වාමින් වහන්සේ නමක්/ ආගමික පූජකවරයෙක්)						

Person/ Service<br>(පුද්ගලයා / සේවාව)		Very helpful<br>ඉතාමත්ම උපකාරී වේ	Fairly helpful<br>තරමක් උපකාරී වේ	Neither helpful nor unhelpful<br>උපකාරී හෝ අනුපකාරී නොවේ	Fairly unhelpful<br>තරමක් අනුපකාරී වේ	Very unhelpful<br>ඉතාමත්ම අනුපකාරී වේ	Don't know<br>දන්නේ නැත
13)	Ayurveda practitioner/ practitioner of indigenous treatments<br>(ආයුර්වේද වෛද්‍යවරයෙක්/ දේශීය ප්‍රතිකාර සපයන්නෙක්)						
14)	Spiritual healer<br>(අධ්‍යාත්මික සුව කරන්නෙක්)						
15)	An astrologer<br>(ජ්‍යොතිෂ්‍යවේදියෙක්)						
16)	A university lecturer<br>(විශ්ව විද්‍යාල කථිකාචාර්යවරයෙක්)						
17)	Faculty Personal Tutor<br>(පීඨයෙන් පත් කර ඇති පෞද්ගලික ප්‍රශ්නවලට යොමු විය යුතු කථිකාචාර්යවරයා)						
18)	A university student counsellor<br>(විශ්ව විද්‍යාල ශිෂ්‍ය උපදේශකවරයෙක්)						
19)	A university medical officer<br>(විශ්ව විද්‍යාල වෛද්‍යවරයෙක්)						
20)	Sub-warden in hostel<br>(නේවාසිකාගාර උප පාලකවරයා)						
21)	A mental health professional at the University Psychiatry Unit<br>(විශ්ව විද්‍යාල මනෝ වෛද්‍ය ඒකකයේ මානසික සෞඛ්‍ය වෘත්තීයවේදියෙක්)						
22)	Not approach anyone for help and deal with problem alone<br>(උපකාර සඳහා කිසිවෙකු වෙත යොමු නොවී තනිවම ප්‍රශ්නයට මුහුණදීම)						

14. Given below are some actions/ interventions 'Z' might engage in when trying to deal with this problem. Indicate how helpful/ unhelpful you think they would be.

(මෙම ප්‍රශ්නයට මුහුණදීමේදි 'Z' නියැලිය හැකි ක්‍රියාවන්/පිලියම් පහත දක්වා ඇත. ඔබේ මතය අනුව ඒවා කොපමණ උපකාරී වේද/නොවේද යන්න දක්වන්න.)

Interventions<br>මැදිහත්වීම්		Very helpful<br>ඉතාමත්ම උපකාරී වේ	Fairly helpful<br>තරමක් උපකාරී වේ	Neither helpful nor unhelpful<br>උපකාරී හෝ අනුපකාරී නොවේ	Fairly unhelpful<br>තරමක් අනුපකාරී වේ	Very unhelpful<br>ඉතාමත්ම අනුපකාරී වේ	Don't know<br>දන්නේ නැත
1)	Become more active in daily activities<br>(දෛනික කටයුතුවලදී පෙරට වඩා ක්‍රියාශීලි වීම)						
2)	Do physical exercise<br>(ශාරීරික ව්‍යායාම කිරීම)						
3)	Do activities he/ she enjoys<br>(ප්‍රියකරන ක්‍රියාකාරකම් කිරීම)						
4)	Stop going to university and stay at home<br>(විශ්ව විද්‍යාලයට යාම නවතා නිවසේ නැවතීම)						
5)	Perform religious activities<br>(ආගමික කටයුතු කිරීම)						
6)	Take a vow<br>(භාරයක් වීම)						
7)	Perform exorcism rituals<br>(බලි තොවිල් කර්මයක් කිරීම)						
8)	Get advice from close associates<br>(හිතවත් අයගෙන් උපදෙස් ගැනිම)						
9)	Get counselling or psychological therapy<br>(උපදේශනය හෝ මනෝවිද්‍යාත්මක ප්‍රතිකාර ලබාගැනිම)						
10)	Take western medicine to improve mood<br>(මනෝභාවය යහපත් කිරීමට බටහිර ඖෂධ ගැනීම)						
11)	Take non-western medicines<br>(බටහිර නොවන ඖෂධ ගැනීම)						

	Interventions<br>මැදිහත්වීම්	Very helpful<br>ඉතාමත්ම උපකාරි වේ	Fairly helpful<br>තරමක් උපකාරි වේ	Neither helpful nor unhelpful<br>උපකාරි හෝ අනුපකාරි නොවේ	Fairly unhelpful<br>තරමක් අනුපකාරි වේ	Very unhelpful<br>ඉතාමත්ම අනුපකාරි වේ	Don't know<br>දන්නේ නැත
12)	Wear blessed items such as talismans<br>(සුර වැනි ආශිර්වාද කර ඇති දේ පැළඳීම)						
13)	Perform ceremonies to expel the 'evil eye'<br>(ඇස් වහ කට වහ නැති කිරීමට පිලිවෙත් කිරීම)						
14)	Do meditation, yoga or other relaxation exercises<br>(භාවනා, යෝග හෝ වෙනත් සිත සැහැල්ලු කරන ව්‍යායාම් කිරීම)						
15)	Do Stress Management courses<br>(ආතති කළමණාකරණ පාඨමාලාවල නියැලීම)						
16)	Improve eating habits<br>(හොඳ ආහාර පුරුදු ඇති කිරීම)						
17)	Improve sleeping habits<br>(නිදා ගැනීමේ පුරුදු දියුණු කිරීම)						
18)	Get information from internet about dealing with problem<br>(ප්‍රශ්නයට මුහුණ දීම පිළිබඳ තොරතුරු අන්තර්ජාලයෙන් ලබාගැනීම)						
19)	Read books on dealing with problem<br>(ප්‍රශ්නයට මුහුණ දීම පිළිබඳ පොත් කියවීම)						
20)	Talk to others who have faced similar problems<br>(මේ හා සමාන ප්‍රශ්නවලට මුහුණදුන් අයට කතා කිරීම)						
21)	Use alcohol/ cigarettes/ drugs<br>(මධ්‍යසාර/සිගරට්/මත්ද්‍රව්‍ය භාවිතා කිරීම)						
22)	Cut down use of alcohol/ cigarettes/ drugs<br>(මධ්‍යසාර/ සිගරට්/මත්ද්‍රව්‍ය භාවිතය අඩු කිරීම)						

Interventions මැදිහත්වීම්		Very helpful ඉතාමත්ම උපකාරී වේ	Fairly helpful තරමක් උපකාරී වේ	Neither helpful nor unhelpful උපකාරී හෝ අනුපකාරී නොවේ	Fairly unhelpful තරමක් අනුපකාරී වේ	Very unhelpful ඉතාමත්ම අනුපකාරී වේ	Don't know දන්නේ නැත
23)	Get hypnosis (මෝහනයට යොමු වීම)						
24)	Get Electro Shock therapy (විද්‍යුත් කම්පන ප්‍රතිකාරයට යොමු වීම)						
25)	Take medicine for physical problems (කායික ගැටලුවලට ඖෂධ ගැනීම)						
26)	Take pain killers (eg: Paracetamol) (වේදනානාශක ගැනීම (උදා: පැරසිටමෝල්))						
27)	Take vitamins (විටමින් ගැනීම)						
28)	Take sleeping tablets (නිදි පෙති ගැනිම)						

15. Below are statements about 'Z's problem. Indicate how much you agree or disagree with each statement.
('Z' ගේ ප්‍රශ්නය පිළිබඳ ප්‍රකාශ පහත දැක්වේ. එක් එක් ප්‍රකාශය සමඟ ඔබ එකඟවන හෝ නොවන ප්‍රමාණය දක්වන්න)

Statement (ප්‍රකාශය)		Strongly agree (දැඩි ලෙස එකඟ වෙමි)	Agree (එකඟ වෙමි)	Neither agree nor disagree (එකඟ හෝ නොඑකඟ නොවෙමි)	Disagree (නොඑකඟ වෙමි)	Strongly Disagree (දැඩි ලෙස නොඑකඟ වෙමි)
1)	'Z' could make the problem just go away if 'Z' wanted to. ('Z'ට උවමනා නම් ඉබේම ප්‍රශ්නය නැති කර ගත හැක.)					
2)	'Z's problem is a sign of personal weakness. ('Z'ගේ ප්‍රශ්නය 'Z'ගේ පෞද්ගලික දුර්වලතාවයේ ලක්‍ෂණයකි.)					
3)	'Z's problem is not a real illness. ('Z'ගේ ප්‍රශ්නය සැබෑ රෝගයක් නොවේ.)					
4)	'Z' could be dangerous to others. ('Z' අනෙක් අයට අනතුරුදායක විය හැකිය.)					

	Statement (ප්‍රකාශය)	Strongly agree (දැඩි ලෙස එකඟ වෙමි)	Agree (එකඟ වෙමි)	Neither agree nor disagree (එකඟ හෝ නොඑකඟ නොවෙමි)	Disagree (නොඑකඟ වෙමි)	Strongly Disagree (දැඩි ලෙස නොඑකඟ වෙමි)
5)	It is best to avoid 'Z' so that you don't develop this problem yourself. (මෙම ප්‍රශ්නය ඔබ තුළත් වර්ධනයවීම වැළැක්වීමට 'Z'ව මග හැරීම හොඳම පිළිවෙතයි.)					
6)	'Z's problem makes 'Z's behaviour unpredictable. ('Z'ගේ ප්‍රශ්නය 'Z'ගේ හැසිරීම අවිනිශ්චිත කරයි.)					
7)	You would not tell anyone if you had a problem like 'Z's ('Z'ගේ ප්‍රශ්නය ඔබට තිබේ නම් ඔබ එය කිසිවෙකුට කියන්නේ නැත.)					

16. Indicate how willing you are to do the activities below with 'Z'.

(පහත ඇති ක්‍රියාකාරකම් 'Z' සමඟ කිරීමට ඔබ කොපමණ කැමැති වන්නේ ද යැයි දක්වන්න)

	Would you be willing to do the following? (ඔබ පහත දේ කිරීමට කැමැති වන්නේ ද?)	Yes, definitely (ඔව්, අනිවාර්යයෙන්ම)	Yes, probably (ඔව්, බොහෝ දුරට)	Probably not (බොහෝ දුරට නැහැ)	Definitely not (අනිවාර්යයෙන්ම නැහැ)
1)	go out with 'Z' in the weekend ('Z' සමඟ සති අන්තයේ එළියට යාම)				
2)	do joint study with 'Z' ('Z' සමඟ එකට පාඩම් කිරීම)				
3)	invite 'Z' to your house ('Z' ට ඔබේ නිවසට ආරාධනා කිරීම)				
4)	go to 'Z's house ('Z' ගේ නිවසට යාම)				
5)	develop a close friendship with 'Z' ('Z' සමඟ සමීප මිත්‍රත්වයක් ගොඩනැගීම)				

17. Has anyone in your family or close circle of friends had a problem like 'Z's? (excluding yourself) (ඔබේ පවුලේ කිසිවෙකුට හෝ ඔබේ සමීප මිතුරෙකුට හෝ 'Z' ට සමාන ප්‍රශ්නයක් තිබී ඇත්ද? (ඔබ හැර))

Yes ☐ (ඔව්)   No ☐ (නැත)   Don't know ☐ (දන්නේ නැත)

18. Have you ever had a problem like ‘Z’? (ඔබට ‘Z’ ට සමාන ප්‍රශ්නයක් තිබී ඇත්ද?)

Yes ☐ → Go to 19 / 19 ට යන්න (ඔව්)

No ☐ → Go to 20 / 20 ට යන්න (නැත)

Don’t know ☐ → Go to 19 / 19 ට යන්න (දන්නේ නැත)

19. Did you deal with the problem on your own, without getting help from others? (අන් අයගෙන් උපකාර නොගෙන ඔබ එම ප්‍රශ්නයට තනිවම මුහුණදුන්නේද?)

Yes ☐ (ඔව්)

Tried first but got help later ☐ (මුලින් එසේ තැත් කළත් පසුව උපකාර ලබා ගත්තා)

No ☐ (නැත)

20. Indicate how often you have been bothered by the following problems during the past two weeks. (පසුගිය සති දෙක තුළ ඔබ පහත ප්‍රශ්න නිසා කොයි තරම් දින ගණනක් පීඩා වින්දාද යැයි දක්වන්න)

**Over the last 2 weeks, how often have you been bothered by any of the following problems?** (පසුගිය සති දෙක ඇතුළත පහත එක් එක් ප්‍රශ්නය නිසා ඔබ කොයි තරම් දින ගණනක් පීඩා වින්දා ද?)		**Not at all** (කිසිසේත්ම නැහැ)	**Several days** (දින කීපයක්)	**More than half the days** (දින වැඩි හරියක දී)	**Nearly every day** (හැමදාම වගේ)
1)	Little interest or pleasure in doing things (වැඩ කිරීමේ සතුට හෝ උනන්දුව අඩුකම)				
2)	Feeling down, depressed, or hopeless (හිතේ දුක්බර, බලාපොරොත්තු රහිත, බිඳ වැටුණු හෝ කලකිරුණු හැඟීම)				
3)	Trouble falling or staying asleep, or sleeping too much (නින්දට යාමේ අපහසුතාව හෝ නින්ද නිතර කැඩෙන බව, එහෙම නැත්නම් ඕනෑවට වඩා නින්ද යාම)				
4)	Feeling tired or having little energy (තෙහෙට්ටු ගතිය හෝ ඇඟේ පණ නැති ගතිය)				
5)	Poor appetite or overeating (කෑම අරුචිය, එහෙම නැත්නම් උවමනාවට වඩා කෑම ගැනීම)				
6)	Feeling bad about yourself- or that you are a failure or have let yourself or your family down (තමා ගැන කලකිරීම හෝ තමා අසාර්ථක කෙනෙකු ලෙස සිතීම, නැතහොත් තමාගේ හෝ තම පවුලේ අයගේ අපේක්ෂාවන් ඔබ නිසා බිඳ වැටුණු බව හැඟීම)				
7)	Trouble concentrating on things, such as reading the newspaper or watching television (පත්තර කියවීම හෝ රූපවාහිනිය බැලීම වැනි දේවලදී හිත එක තැනක තියාගෙන ඉන්න අපහසු වීම)				

Over the last 2 weeks, how often have you been bothered by any of the following problems? (පසුගිය සති දෙක ඇතුළත පහත එක් එක් ප්‍රශ්නය නිසා ඔබ කොයි තරම් දින ගණනක් පීඩා වින්දා ද?)		Not at all (කිසිසේත්ම නැහැ)	Several days (දින කීපයක්)	More than half the days (දින වැඩි හරියක දී)	Nearly every day (හැමදාම වගේ)
8)	Moving or speaking so slowly that other people could have noticed. Or the opposite - being so fidgety or restless that you have been moving around a lot more than usual (වෙන අයගෙ අවධානය යොමු වෙන්න පුළුවන් තරමට, ඔබගේ ගමන් කරන හෝ කථා කරන වේගය අඩු වීම, එහෙම නැත්නම් ඒකෙ අනික් පැත්ත- ඔබ කොයිතරම් අසහනකාරී හෝ නොසන්සුන් වුවා ද කිව්වොත් ඔබ සාමාන්‍ය ප්‍රමාණයට වඩා එහා මෙහා ගමන් කිරීම)				
9)	Thoughts that you would be better off dead, or of hurting yourself (මැරුණා නම් මීට වඩා හොඳයි කියා සිතීම හෝ ජීවිතයට හානියක් කර ගන්න සිතීම)				

21. Indicate if you faced any of the following situations in the past year, and if so, how upset you became by them. If some of these occurred more than a year ago, but you are still upset because of them, rate how upset you have been in the past year.

(පසුගිය වසර තුල ඔබ පහත සිද්ධීන්වලට මුහුණ දුන්නේද යන්නත්, එසේ නම් ඒවා ඔබව කොතරම් කැලඹීමකට පත්කළේද යන්නත් දක්වන්න. සමහර සිදුවීම් වසරකට පෙර සිදුවුවත්, ඒවා නිසා ඔබ තවමත් කැලඹීමෙන් පසුවන්නේනම්, පසුගිය වසර තුල ඒවා නිසා ඔබ කැලඹීමෙන් පසුවූ තරම දක්වන්න)

Situation (අවස්ථාව)		Did not happen (සිදු නොවුනි)	Happened- not upsetting (සිදු වුනි- කැලඹීමට පත්නොවුනි)	Happened- moderately upsetting (සිදුවුනි- සාමාන්‍ය ලෙස කැලඹීමට පත්වුනි)	Happened- extremely upsetting (සිදුවුනි- දැඩි ලෙස කැලඹීමට පත්වුනි)	Not relevant (අදාල නොවේ)
1)	You yourself suffered a serious illness, injury or an assault (ඔබ දරුණු රෝගයකින්, බරපතල තුවාලයකින් හෝ පහරකෑමකින් පීඩා වින්දා)					
2)	A serious illness, injury or assault happened to a close relative (සමීප ඥාතියෙකු දරුණු රෝගයකට, බරපතල තුවාලයකට හෝ පහරකෑමකට ලක්වුවා)					
3)	Your parent, sibling, child or spouse died (ඔබේ දෙමව්පියෙක්,සහෝදරයෙක්, දරුවෙක් හෝ සැමියා/බිරිඳ මියගියා)					

**Situation** (අවස්ථාව)		**Did not happen** (සිදු නොවුනි)	**Happened-not upsetting** (සිදු වුනි- කැලඹීමට පත්නොවුනි)	**Happened-moderately upsetting** (සිදුවුනි- සාමාන්‍ය ලෙස කැලඹීමට පත්වුනි)	**Happened-extremely upsetting** (සිදුවුනි- දැඩි ලෙස කැලඹීමට පත්වුනි)	**Not relevant** (අදාල නොවේ)
4)	A friend or another close relative (aunt, cousin, grandparent) died<br>(සමීප මිතුරෙක් හෝ වෙනත් සමීප ඥාතියෙක් (නැන්දා,ඥාති සොයුරා,සීයා හෝ ආච්චි මියගියා))					
5)	You or your parents or a very close family member had a separation due to marital difficulties<br>(ඔබ හෝ ඔබේ දෙමව්පියන් හෝ බොහෝ සමීප ඥාතියෙකු විවාහ දුෂ්කරතා නිසා වෙන් වූවා)					
6)	You broke off a steady relationship<br>(ඔබට තිබූ දැඩි ප්‍රේම සම්බන්ධතාවයක් කැඩී ගියා)					
7)	You had a serious problem with a close friend, neighbour or relative<br>(සමීප මිතුරෙකු, අසල්වැසියෙකු හෝ ඥාතියෙකු සමඟ ඔබට බරපතල ප්‍රශ්නයක් ඇති වූවා)					
8)	You had problems with your studies or got unsatisfactory results at exams<br>(ඔබට අධ්‍යාපන කටයුතු සමග අපහසුතා ඇති වූවා හෝ ඔබ විභාගවලදී අසතුටුදායක ප්‍රතිඵල ලබා ගත්තා)					
9)	You became unemployed or you were seeking work unsuccessfully for more than one month<br>(ඔබේ රැකියාව අහිමි වූවා හෝ මාසයකට වැඩි කාලයක් රැකියාවක් සෙවීමේ අසාර්ථක උත්සාහයක යෙදුණා)					
10)	You were sacked from your job<br>(ඔබව රැකියාවෙන් නෙරපා හැරියා)					
11)	You or your family had financial problems<br>(ඔබට හෝ ඔබේ පවුලට ආර්ථික ප්‍රශ්න ඇති වූවා)					
12)	You had a court appearance and/ or problems with the police<br>(ඔබට උසාවියේ පෙනී සිටීමට සිදුවූවා සහ/හෝ පොලීසිය සමඟ ප්‍රශ්න ඇති වූවා)					

	Situation (අවස්ථාව)	Did not happen (සිදු නොවුනි)	Happened- not upsetting (සිදු වුනි- කැලඹීමට පත්නොවුනි)	Happened- moderately upsetting (සිදුවුනි- සාමාන්‍ය ලෙස කැලඹීමට පත්වුනි)	Happened- extremely upsetting (සිදුවුනි- දැඩි ලෙස කැලඹීමට පත්වුනි)	Not relevant (අදාල නොවේ)
13)	Something you valued was lost or stolen<br>(ඔබ අගය කල යමක් නැති වූවා හෝ සොරකම් කර තිබුණා)					
14)	You were harassed (verbally, mentally, physically or sexually) by another student at university<br>(ඔබ විශ්ව විද්‍යාලයේ වෙනත් සිසුවෙකු අතින් හිරිහැරයකට ලක් වූවා(වාචිකව, මානසිකව, ශාරීරිකව හෝ ලිංගිකව))					
15)	You were harassed (verbally, mentally, physically or sexually) by a staff member at university<br>(ඔබ විශ්ව විද්‍යාලයේ කාර්ය මණ්ඩල සාමාජිකයෙකු අතින් හිරිහැරයකට ලක් වූවා(වාචිකව, මානසිකව, ශාරීරිකව හෝ ලිංගිකව))					
16)	You experienced a natural disaster or its resultant effects (eg: injury, death of loved ones, damage to property, displacement)<br>(ඔබ ස්වභාවික ආපදාවක් අත් වින්දා හෝ එහි ප්‍රතිවිපාකවලට ලක් වූවා (උදා:බරපතල තුවාල, සමීපතමයින්ගේ මරණ,දේපළ හානි, අවතැන්වීම්))					
17)	You were exposed to the negative effects of war, armed conflict or terrorism (eg: injury, death of loved ones, damage to property, displacement)<br>(ඔබ යුද්ධය, අවි ගැටුම් හෝ ත්‍රස්තවාදයේ ඍණාත්මක ප්‍රතිවිපාකවලට ලක් වූවා (උදා:බරපතල තුවාල,සමීපතමයින්ගේ මරණ, දේපළ හානි, අවතැන්වීම්))					
18)	You witnessed or were subjected to violence within your family<br>(ඔබ පවුල තුළ ප්‍රචණ්ඩත්වය සියැසින්ම දුටුවා හෝ එයට ගොදුරු වූවා)					
